# Criteria for evaluating risk prediction of multiple outcomes

**DOI:** 10.1177/0962280220929039

**Published:** 2020-06-29

**Authors:** Frank Dudbridge

**Affiliations:** Department of Health Sciences, University of Leicester, Leicester, UK

**Keywords:** Risk prediction, multiplicity, screening, multivariate analysis, biomarkers, polygenic risk score

## Abstract

Risk prediction models have been developed in many contexts to classify individuals according to a single outcome, such as risk of a disease. Emerging “-omic” biomarkers provide panels of features that can simultaneously predict multiple outcomes from a single biological sample, creating issues of multiplicity reminiscent of exploratory hypothesis testing. Here I propose definitions of some basic criteria for evaluating prediction models of multiple outcomes. I define calibration in the multivariate setting and then distinguish between outcome-wise and individual-wise prediction, and within the latter between joint and panel-wise prediction. I give examples such as screening and early detection in which different senses of prediction may be more appropriate. In each case I propose definitions of sensitivity, specificity, concordance, positive and negative predictive value and relative utility. I link the definitions through a multivariate probit model, showing that the accuracy of a multivariate prediction model can be summarised by its covariance with a liability vector. I illustrate the concepts on a biomarker panel for early detection of eight cancers, and on polygenic risk scores for six common diseases.

## 1 Introduction

Risk prediction is important in many medical contexts in which prediction models can guide decision making.^1^ Examples include primary prevention, such as cholesterol reduction in subjects at risk of cardiovascular disease^2^; secondary prevention, such as the targeted enrolment of individuals into screening programmes^3^; allocation of treatment according to prognosis^4^; and differential diagnosis.^5^ In general, models are constructed with the prediction of a single discrete outcome in mind. Thus models for identifying individuals at risk of, for example, breast cancer,^6^ cardiovascular disease^7^ and diabetes^8^ have been developed by separate research communities with different study cohorts, although the models may share some variables and identify some of the same individuals as at risk. Consequently, evaluation of prediction models is also done according to single outcomes.

The emergence of “-omic” and other molecular biomarkers has raised the prospect of panels of features that can simultaneously predict multiple outcomes from a single biological sample. For example, a blood test called CancerSEEK has been proposed for early detection of eight cancers from circulating proteins and tumour DNA mutations.^9^ Genome-wide association studies have garnered particular attention as many diseases are heritable and the DNA sequence is fixed throughout life. Because many diseases are influenced by numerous variants across the entire genome, genetic risk can be efficiently measured with a generic micro-array,^10^ and in principle could be calculated for multiple conditions at any point in life. Epigenetic variation may also provide useful risk stratification and has been advocated for the early detection of several cancers.^11^ Furthermore, the emergence of large, broadly phenotyped cohorts such as UK Biobank^12^ provides useful resources for developing and evaluating such models.

Apart from the practical efficiencies of conducting several assessments in parallel, simultaneous prediction has other potentially useful applications. Individuals may be more concerned about their risk across a range of conditions rather than of one in particular, a demand increasingly targeted by direct-to-consumer genetic testing companies.^13^ Furthermore, some interventions may be effective for several conditions, and identification of individuals at increased risk of any of them may lead to greater impact of such interventions. As a simple example, body mass index is associated with several diseases with otherwise distinct causes, including coronary heart disease, type-2 diabetes, breast cancer and depression.^14^ A weight loss intervention might be more effective when targeted to those at increased risk of any of those conditions. Similarly, evidence that aspirin usage could reduce the risk of various cancers^15^ as well as of cardiovascular disease suggests that risk prediction for a set of diseases could be of benefit. More speculatively, forensic applications could utilise simultaneous prediction of phenotypes from anonymous DNA samples.^16,17^

Prediction of this nature is already done informally using recurrent risk factors such as age, gender, smoking and blood pressure. For example, in the UK the NHS Health Check is offered to individuals aged between 40 and 74 on account of the strong association of age with risk of stroke, kidney disease, heart disease, type 2 diabetes and dementia. For such risk factors, their strength of association and ease of measurement obviate any need for formal evaluation over many outcomes. But for emerging risk factors it is less clear whether their utility is enhanced by their potential to predict multiple outcomes. There are problems of multiplicity reminiscent of those in exploratory hypothesis testing, but a framework is lacking for addressing these issues in the context of risk prediction.

Prediction of multiple outcomes can be distinguished from prediction of a single composite outcome. Composite outcomes have been used to group related conditions, such as cardiovascular disease,^18^ and to define outcomes of specific interest such as frailty and all-cause mortality.^19^ Prediction of such outcomes may be viewed as a crude form of multiple outcome prediction: here I consider the composite evaluation of multiple predictions, rather than the evaluation of a single composite prediction. Composite evaluation may offer improved accuracy over a composite outcome; pragmatically it can use predictors developed individually for each outcome without the need to develop a specific predictor for their composite.

Several authors have studied the statistical modelling of a multivariate response, using methods such as partial least squares^20^ and multivariate linear regression.^21–23^ While it is recognised that prediction can be improved by exploiting correlation among responses, the literature has emphasised methods to improve model fitting, with accuracy typically measured by squared error metrics for each response marginally^22,24^ or in total across responses.^21^ This may be adequate in applications such as chemometrics and genetic selection where the responses are quantitative, but is less satisfying for prediction of discrete outcomes. Here I am not concerned with model fitting *per se* but in evaluating models, however estimated, in the context of their joint risk predictions. There is some work on mutually exclusive events, such as polytomous outcomes^25^ and competing risks,^26^ but general vectors of dichotomous outcomes have not been studied.

Here I propose definitions of some basic criteria for evaluating risk prediction models of multiple outcomes. The evaluation of single outcome models, while not a settled question, has at least a standard set of core criteria that serve as a basis for more nuanced assessment.^27^ The present aim is to propose a similar set of core criteria as a starting point for the development of more refined approaches. I do not aim to give a complete account of multiple outcome prediction, but to identify and open discourse around some basic issues in this emerging area.

In section 2, I identify four senses in which multiple predictions can be evaluated, termed outcome-wise, joint, and weak and strong panel-wise. Examples are given in which each sense of prediction may be appropriate. I define sensitivity, specificity, concordance, and relative utility in each of these senses. In section 3, I develop analytical expressions for each of these quantities from a multivariate probit model. These show that the accuracy of a multivariate prediction model can be summarised by its covariance with a liability vector, and from this covariance matrix all the proposed criteria can be derived. Section 4 applies the results to some examples of current applications, and uses the model of section 3 to project their future performance as improved predictors are developed. Section 5 provides some discussion.

## 2 Definitions

### 2.1 Preliminaries

For individual i=1,…,N, let $D_{i}\in {\left\{0,1\right\}}^{m}$ be a vector of binary indicators for m dichotomous outcomes. Say that outcome j *did occur* when the j-th element of $D_{i}$ is 1, and the outcome *did not occur* when that element is 0. Similarly to Gail and Pfeiffer,^28^ define the vector $\pi_{i}$ whose j-th component is the probability of outcome j in individual i. Where necessary, components are identified by brackets: for example, $\pi_{i[j]}$ denotes the j-th component of $\pi_{i}$. Let $X_{i}$ be a vector of predictors and consider a *marginal risk prediction model* r(x) as a mapping from the set Ω of possible values of $X_{i}$ to ${\left[0,1\right]}^{m}$. The model is understood as marginal in that, reflecting much current practice, r(x) provides a risk prediction for each outcome but not for combinations of outcomes. In particular, correlations between outcomes may arise from comorbidity, competing risks or other sources, so that outcome-specific predictions may not be easily combined into predictions for groups of outcomes.

As for single outcome prediction, calibration is a desirable property of a risk predictor, and it will be generally useful for the predictor to be calibrated for all outcomes. Informally, calibration requires that predicted risks equal actual risks, but a distinction can be made between the risk among individuals with given predictors x, and risk among individuals with given predictions r(x). These quantities may differ if r(x) has the same value for many values of x, as in the case of a risk score formed as a linear combination of many predictors.^29^

**Definition 1**

*The risk prediction model* r(x)* is strongly calibrated* *if***** E(D|x)=E(π|x)=r(x) *for all***** x∈Ω. *The predictor is weakly calibrated* *if* ****$E\left(D\text{|}r\left(x\right)=r^{\text{*}}\right)=E(\pi |r\left(x\right)=r^{\text{*}})=r^{\text{*}}$ *for all* x∈Ω *and* $r^{\text{*}}\in {\left[0,1\right]}^{m}$.

Calibration is usually assessed by plots or goodness-of-fit tests.^29–31^ While these approaches could generalise to a multivariate setting, the following component-wise definition is sufficient for application to marginal prediction models, and can be assessed by applying univariate methods to each component of r(x).

**Definition 2**

*The risk prediction model* r(x) *is strongly component-wise calibrated if *$E\left(D_{\left[j\right]}\text{|}x\right)=E\left(\pi_{\left[j\right]}\text{|}x\right)=r_{\left[j\right]}\left(x\right)$ *for all* j=1,⋯,m *and***** x∈Ω. *The prediction model is* *weakly component-wise calibrated* *if***** $E\left(D_{\left[j\right]}\text{|}r_{\left[j\right]}\left(x\right)=r^{\text{*}}\right)=E(\pi_{\left[j\right]}|r_{\left[j\right]}\left(x\right)=r^{\text{*}})=r^{\text{*}}$ *for all*****j=1,⋯,m, x∈Ω *and***** $r^{\text{*}}\in [0,1]$.

Calibration implies component-wise calibration, but the converse need not hold. In the rest of the paper I assume that r(x) is at least weakly component-wise calibrated.

Let $t\in {\left[0,1\right]}^{m}$ be a vector of risk thresholds. Each individual i is assigned to a high-risk category for each outcome j where $r_{\left[j\right]}(X_{i})\geq t_{\left[j\right]}$.

### 2.2 Outcome-wise criteria

A straightforward approach is to treat outcomes, rather than individuals, as the sampling units and then apply standard criteria to the vectorised outcomes. Such a view might be appropriate when the consequences of predicting or developing the outcomes are independent. This approach has been used in evaluating carrier screening panels for Mendelian disorders.^32^ Another example might be in molecular screening for allergies.^33^

**Definition 3**

*Outcome-wise sensitivity is the probability of a positive prediction for an outcome that did occur. Over the joint sample space of* D *and* ***X***

$$sens_{O}(t)=\frac{E_{D,X}\left[D^{\text{′}}I(r(X)\geq t)\right]}{E_{D}\left[D^{\text{′}}1\right]}$$

where I is a vector of component-wise indicators and 1 is the vector with all elements equal to one. This is equivalent to the classical sensitivity when m=1. However, whereas the classical sensitivity does not depend on the outcome probability E(π), the outcome-wise sensitivity does depend on the relative outcome probabilities. To see this, write

sensO(t)=ED,X[∑jD[j]I(r[j](X)≥t[j])]ED[D′1]=∑j=1mED,X[D[j]I(r[j](X)≥t[j])]ED[D′1]=∑j=1mED,X[D[j]I(r[j](X)≥t[j])]ED[D[j]]ED[D[j]]ED[D′1]=∑j=1mPr(r[j](X)≥t[j]|D[j]=1)Pr(D[j]=1)∑kPr(D[k]=1)

The first term in the summand is the classical sensitivity for outcome j, so the outcome-wise sensitivity is the weighted sum of the individual outcome sensitivities, with the weights as the relative outcome probabilities. Therefore, a sample estimate of outcome-wise sensitivity may be subject to ascertainment bias, but a population estimate may be obtained by weighting the individual outcome sensitivities using external estimates of outcome probabilities.

Weights may be used to attach greater importance to the prediction of some outcomes. This may be done by generalising the outcome-wise sensitivity to

$$sens_{O}(t)=\frac{E_{D,X}\left[D^{\text{′}}\mathrm{WI}(r(X)\geq t\right)]}{E_{D}\left[D^{\text{′}}W1\right]}$$

where W is a diagonal matrix with positive entries. Again this is equivalent to a weighted sum of individual outcome sensitivities, with the weights as the relative outcome probabilities scaled by the respective diagonal elements of W.

**Definition 4**

*Outcome-wise specificity is the probability of a negative prediction for an outcome that did not occur.*

$$spec_{O}(t)=\frac{E_{D,X}\left[{\left(1-D\right)}^{\text{′}}I(r\left(X\right)<t\right)]}{E_{D}\left[{\left(1-D\right)}^{\text{′}}1\right]}$$

Similarly to the sensitivity, the outcome-wise specificity is the weighted sum of the individual outcome specificities, with the weights as the relative probabilities of the complementary outcomes. General weights may be introduced as for the sensitivity.

A standard, if often criticised^34–36^ summary of sensitivity and specificity is the area under the receiver operating characteristic (ROC) curve, which for a single outcome is constructed by plotting sensitivity against 1-specificity over the range of t. The idea of a ROC does not easily generalise to multiple outcomes because vectors t need not parameterise a one-to-one mapping of specificity to sensitivity. However, the C- (concordance) index,^37^ which for a single outcome is equivalent to the area under the entire ROC curve, can be extended more readily.

The C-index for a single outcome is the probability that, given one individual with the outcome and one without, the prediction is higher for the former, i.e. $\text{Pr}(r\left(X_{i_{1}}\right)>r(X_{i_{o}})|D_{i_{1}}=1,D_{i_{o}}=0)$. An outcome-wise extension might be to evaluate the same probability over outcomes rather than individuals. However, this would compare the predicted risk for an outcome that did occur to the predicted risk of a different outcome that did not occur, which is difficult to interpret when the elements of t are unequal. Stated differently, if the aim is to quantify how well r(x) discriminates outcomes that did occur from those that did not, it makes little sense to compare predictions for different outcomes when the risk thresholds for those outcomes may be different.

A more satisfactory approach is to compare a prediction for an outcome that did occur to a prediction for *the same* outcome when it did not occur. This just yields the C-index for that outcome, so the expected C-index for multiple outcomes is the weighted sum of individual outcome C-indices. For outcome j the probability of observing a discordant pair of outcomes is $E\left(D_{\left[j\right]}\right)\left(1-E\left(D_{\left[j\right]}\right)\right)$ giving

**Definition 5**

*Outcome-wise C-index is the weighted sum of individual outcome C-indices.*

$$\overset{m}{\underset{j=1}{\sum }}\text{Pr}(r_{\left[j\right]}\left(X_{i_{1}}\right)>r_{\left[j\right]}(X_{i_{0}})|D_{i_{1}[j]}=1,D_{i_{o}[j]}=0)\frac{E(D_{\left[j\right]})(1-E\left(D_{\left[j\right]}\right))}{\sum_{k}E(D_{\left[k\right]})(1-E\left(D_{\left[k\right]}\right))}$$

One criticism of the ROC curve is that it treats sensitivity and specificity equally when they may entail different benefits and costs. The relative utility curve has been proposed to address this issue,^38,39^ and is especially useful for comparing different risk prediction models. Here I summarise its derivation for one outcome before developing an outcome-wise extension.

Let b be the benefit of correctly predicting an outcome that did occur, and c the cost of incorrectly predicting an outcome that did not occur. Given a decision making risk threshold t, for an individual i with risk prediction $r\left(X_{i}\right)=t$ the net benefit of a positive prediction is $b\mathrm{Pr}\left(D_{i}=1\text{|}r\left(X_{i}\right)=t\right)-c\text{Pr}(D_{i}=0|r\left(X_{i}\right)=t)$ and this is positive when

$$\frac{\;\mathrm{Pr}\left(D_{i}=1\text{|}r\left(X_{i}\right)=t\right)}{\mathrm{Pr}\left(D_{i}=0\text{|}r\left(X_{i}\right)=t\right)}>\frac{c}{b}$$

It follows that if the risk predictor is weakly calibrated, the net benefit is positive if $r(X_{i})>t$ where t is such that

$$\frac{t}{1-t}=\frac{\;\mathrm{Pr}\left(D_{i}=1\text{|}r\left(X_{i}\right)=t\right)}{\;\mathrm{Pr}\left(D_{i}=0\text{|}r\left(X_{i}\right)=t\right)}=\frac{c}{b}$$

Therefore, use of the threshold t implies a cost-benefit ratio of t/(1−t). With this threshold, the expected net benefit over the population is

Pr⁡(r(X)≥t)[bPr⁡(D=1|r(X)≥t)−cPr⁡(D=0|r(X)≥t)] =b[Pr⁡(r(X)≥t|D=1)Pr⁡(D=1)−cbPr⁡(r(X)≥t|D=0)Pr⁡(D=0)] =bPr(D=1)[sens(t)−t1−tPr⁡(D=0)Pr⁡(D=1)(1−spec(t))]

The relative utility is the ratio of this expectation to its theoretical maximum when sensitivity and specificity are both 1, thus

$$RU\left(t\right)=sens\left(t\right)-\frac{t}{1-t}\frac{\mathrm{Pr}\left(D=0\right)}{\mathrm{Pr}\left(D=1\right)}(1-spec\left(t\right))$$

The net benefit is understood as resulting from taking action on a prediction, and so is relative to the result of taking no action. If the default, in the absence of risk prediction, is to take no action, then that is equivalent to a risk predictor with sensitivity 0 and specificity 1 at all thresholds. Conversely, if the default were always to take action then the sensitivity is 1 and the specificity is 0. A default of no action is rational when its relative utility is greater than under the default of always taking action. The definition of RU(t) shows that this occurs when t≥Pr(D=1), termed the *relevant region* for evaluating relative utility.^38^ On the other hand, if the default is to take action, then the analogous definition for t≤Pr(D=1) is

$$RU\left(t\right)=spec\left(t\right)-\frac{1-t}{t}\frac{\mathrm{Pr}\left(D=1\right)}{\mathrm{Pr}\left(D=0\right)}(1-sens\left(t\right))$$

These expressions assume negligible cost of evaluating r(X); more general derivations are provided elsewhere.^38^ Turning now to multiple outcomes, let *b_O_* and *c_O_* represent common values of benefit and cost for all outcomes. (In practice these quantities may vary across outcomes, so they may be thought of here as average values.) Assume that benefits and costs are additive across outcomes within individuals. For an individual i with risk prediction $r\left(X_{i}\right)=t$, the net benefit of a positive prediction is now

$$b_{O}E_{D_{i}}\left(D_{i}^{\text{′}}1\text{|}r\left(X_{i}\right)=t\right)-c_{O}E_{D_{i}}({\left(1-D_{i}\right)}^{\text{′}}1|r\left(X_{i}\right)=t)$$

and is positive when

$$\frac{E_{D_{i}}\left(D_{i}^{\text{′}}1\text{|}r\left(X_{i}\right)=t\right)}{E_{D_{i}}({\left(1-D_{i}\right)}^{\text{′}}1|r\left(X_{i}\right)=t)}>\frac{c_{O}}{b_{O}}$$

If the risk predictor is weakly component-wise calibrated, then

$$E_{D_{i}}\left(D_{i}^{\text{′}}1\text{|}r\left(X_{i}\right)=t\right)=t^{\text{′}}1$$

Therefore, the use of threshold vector t implies the cost–benefit ratio

$$\frac{t^{\text{′}}1}{{\left(1-t\right)}^{\text{′}}1}=\frac{c_{O}}{b_{O}}$$

Under additive benefits and costs, the expected net benefit over the population is

∑j=1mPr⁡(r[j](X)≥t[j])[bOE(D[j]|r[j](X)≥t[j])−cOE(1−D[j]|r[j](X)≥t[j])] =bO[ED,X[D′I(r(X)≥t]−cObOED,X[(1−D)′I(r(X)≥t)]] =bOED[D′1][sensO(t)−t′1(1−t)′1ED[(1−D)′1]ED[D′1](1−specO(t))]

**Definition 6**

*Outcome-wise relative utility for threshold vector* t *is*

$$RU_{O}\left(t\right)=sens_{O}\left(t\right)-\frac{t^{\text{′}}1}{{\left(1-t\right)}^{\text{′}}1}\frac{E_{D}\left[{\left(1-D\right)}^{\text{′}}1\right]}{E_{D}\left[D^{\text{′}}1\right]}(1-spec_{O}\left(t\right))$$

As before, a diagonal weight matrix W can be used to allow some outcomes to contribute more to the relative utility, giving

$$RU_{O}\left(t\right)=sens_{O}\left(t\right)-\frac{t^{\text{′}}W1}{{\left(1-t\right)}^{\text{′}}W1}\frac{E_{D}\left[{\left(1-D\right)}^{\text{′}}W1\right]}{E_{D}\left[D^{\text{′}}W1\right]}(1-spec_{O}\left(t\right))$$

where $sens_{O}\left(t\right)$ and $spec_{O}\left(t\right)$ are also used in their weighted versions. For multiple outcomes, relative utility defines a surface over the space of threshold vectors, $RU:{\left[0,1\right]}^{m}\mapsto (-\infty ,1]$. The relevant region is $\left\{t:t^{\text{′}}1\geq E_{D}\left[D^{\text{′}}1\right]\right\}$ when the default, in the absence of risk prediction, is to take no action for any outcome. When there are outcomes for which the default is to take action, a pragmatic approach is to substitute the complementary outcomes, and thresholds, in the above definitions.

### 2.3 Joint criteria

An issue with outcome-wise measures is that actions are applied to individuals rather than outcomes. In many contexts, it is more appropriate to summarise risk predictions for each individual before taking action. To this end I now define individual-wise measures, which vary according to the definition of a true positive prediction. For joint measures, the aim is to predict the joint occurrence of all outcomes in an individual. An example might be in forensic identification from an anonymous DNA sample, where a profile could be constructed from several traits such as hair colour,^40^ height^16^ and weight,^41^ each discretised into broad categories.

**Definition 7**

*Joint sensitivity is the probability of predicting all outcomes to occur, in an individual for which all outcomes did occur.*

$$sens_{J}\left(t\right)=\text{Pr}(r(X)\geq t|D=1)$$

If the elements of r(X) are jointly independent and the elements of D also are jointly independent, then

sensJ(t)=∏jPr(r[j](X)≥t[j],D[j]=1)∏jPr(D[j]=1)=∏jPr(r[j](X)≥t[j],D[j]=1)Pr(D[j]=1)=∏jPr(r[j](X)≥t[j]|D[j]=1)

In this case, the joint sensitivity is the product of individual outcome sensitivities. However, in the general case of dependence between elements of r(X) or D, the joint sensitivity is not readily expressed in terms of the individual outcome sensitivities.

**Definition 8**

*Joint specificity is the probability of predicting at least one outcome not to occur, in an individual for which at least one outcome did not occur.*

$$spec_{J}\left(t\right)=\text{Pr}(I(r(X)\geq t)\neq 1|D\neq 1)$$

Note that this may depend on the distribution of D and therefore that an estimate of *spec_J_* (***t***) may be subject to ascertainment bias. When information is available on the distribution of D, an unbiased estimate of *spec_J_* (***t***) could be obtained by weighting each observation in which D≠1 by the inverse of its sampling probability.

To define joint concordance, note that D=1⇔min⁡(D)=1 and D≠1⇔min⁡(D)=0.

**Definition 9**

*Joint C-index is the probability that, given one individual in which all outcomes did occur and one individual in which at least one outcome did not occur, the minimum risk prediction is higher in the former individual*.

$$C_{J}=\text{Pr}(\text{min}\left(r\left(X_{i_{1}}\right)\right)>\text{min}\left(r\left(X_{i_{0}}\right)\right)|D_{i_{1}}=1,D_{i_{0}}\neq 1)$$

To define relative utility, let *b_J_* be the benefit of predicting all outcomes to occur when all outcomes did occur, and ${\text{c}}_{\text{J}}$ the cost of predicting all outcomes to occur when at least one outcome did not occur. For an individual i with risk prediction $r\left(X_{i}\right)=t$, the net benefit of a positive prediction is $b_{J}\mathrm{Pr}\left(D_{i}=1\text{|}r\left(X_{i}\right)=t\right)-c_{J}\text{Pr}(D_{i}\neq 1|r\left(X_{i}\right)=t)$ and this is positive when

$$\frac{\;\mathrm{Pr}\left(D_{i}=1\text{|}r\left(X_{i}\right)=t\right)}{\mathrm{Pr}\left(D_{i}\neq 1\text{|}r\left(X_{i}\right)=t\right)}>\frac{c_{J}}{b_{J}}$$

Therefore, use of the threshold vector t implies a cost–benefit ratio of$\;\frac{\;\mathrm{Pr}\left(D_{i}=1\text{|}r\left(X_{i}\right)=t\right)}{\mathrm{Pr}\left(D_{i}\neq 1\text{|}r\left(X_{i}\right)=t\right)}$. With this threshold, the expected net benefit in the population is

Pr⁡(r(X)≥t)[bJPr⁡(D=1|r(X)≥t)−cJPr⁡(D≠1|r(X)≥t)] =bJ[Pr⁡(r(X)≥t|D=1)Pr⁡(D=1)−cJbJPr⁡(r(X)≥t|D≠1)Pr⁡(D≠1)] =bJPr(D=1)[sensJ(t)− Pr⁡(D=1|r(X)=t)Pr⁡(D≠1|r(X)=t)Pr⁡(D≠1)Pr⁡(D=1)(1−specJ(t))]

**Definition 10**

*Joint relative utility for threshold* t *is*

$$RU_{J}\left(t\right)=sens_{J}\left(t\right)-\frac{\;\mathrm{Pr}\left(D=1\text{|}r\left(X\right)=t\right)}{\mathrm{Pr}\left(D\neq 1\text{|}r\left(X\right)=t\right)}\frac{\mathrm{Pr}\left(D\neq 1\right)}{\mathrm{Pr}\left(D=1\right)}(1-spec_{J}\left(t\right))$$

In general Pr⁡(D=1|r(X)=t) must be estimated. As this may be difficult in practice, the following working definition may be useful. If risk predictions and outcomes both are jointly independent, and the risk predictor is weakly component-wise calibrated, then $\mathrm{Pr}\left(D=1\text{|}r\left(X\right)=t\right)=\underset{j}{\prod }t_{\left[j\right]}$ and

$$RU_{J}\left(t\right)=sens_{J}\left(t\right)-\frac{\;\prod_{j}t_{\left[j\right]}}{1-\prod_{j}t_{\left[j\right]}}\frac{\mathrm{Pr}\left(D\neq 1\right)}{\mathrm{Pr}\left(D=1\right)}(1-spec_{J}\left(t\right))$$

The relevant region is {t:Pr⁡(D=1|r(X)=t)≥Pr⁡(D=1)} when the default, in the absence of risk prediction, is to take no action for at least one outcome.

### 2.4 Panel-wise criteria

For panel-wise criteria the aim is to predict the occurrence of at least one outcome in an individual. A correct prediction may, however, be defined in different ways according to whether the predicted outcomes are the same as those that did occur. Here I propose two senses of panel-wise prediction, called the weak and strong senses by analogy to family-wise errors in hypothesis testing.

**Definition 11**

*Weak panel-wise sensitivity is the probability of predicting at least one outcome to occur, in an individual for which at least one outcome did occur*.

$$sens_{S}\left(t\right)=\text{Pr}(I(r(X)\geq t)\neq 0|D\neq 0)$$

The subscript S stands for *screening* as explained later. Note that this may depend on the distribution of D and therefore that an estimate of $sens_{S}\left(t\right)$ may be subject to ascertainment bias. When information is available on the distribution of D, an unbiased estimate of $sens_{S}\left(t\right)$ could be obtained by weighting each observation in which D≠0 by the inverse of its sampling probability.

**Definition 12**

*Weak panel-wise specificity is the probability of predicting no outcomes to occur, in an individual for which no outcomes did occur*.

$$spec_{S}\left(t\right)=\text{Pr}(r\left(X\right)<t|D=0)$$

Definitions 11 and 12 are complementary to the joint sensitivity and specificity, and similarly the weak panel-wise specificity is the product of the component-wise specificities in the case that risk predictions and outcomes both are jointly independent. The complement of weak panel-wise specificity is analogous to the weak sense of family-wise type-1 error rate in hypothesis testing. Similar arguments to the joint criteria give the following definitions of concordance and relative utility.

**Definition 13**

*Weak panel-wise C-index is the probability that, given one individual in which at least one outcome did occur and one individual in which no outcomes did occur, the maximum risk prediction is higher in the former individual*.

$$C_{S}=\text{Pr}(\text{max}\left(r\left(X_{i_{1}}\right)\right)>\text{max}\left(r\left(X_{i_{0}}\right)\right)|D_{i_{1}}\neq 0,D_{i_{0}}=0)$$

**Definition 14**

*Weak panel-wise relative utility for threshold vector* ***t*** *is*

$$RU_{S}\left(t\right)=sens_{S}\left(t\right)-\frac{\;\mathrm{Pr}\left(D\neq 0\text{|}r\left(X\right)=t\right)}{\mathrm{Pr}\left(D=0\text{|}r\left(X\right)=t\right)}\frac{\mathrm{Pr}\left(D=0\right)}{\mathrm{Pr}\left(D\neq 0\right)}(1-spec_{S}\left(t\right))$$

If risk predictions and outcomes both are jointly independent, and the risk predictor is weakly component-wise calibrated, then

$$RU_{S}\left(t\right)=sens_{S}\left(t\right)-\frac{\;1-\prod_{j}(1-t_{\left[j\right]})}{\prod_{j}(1-t_{\left[j\right]})}\frac{\mathrm{Pr}\left(D=0\right)}{\mathrm{Pr}\left(D\neq 0\right)}(1-spec_{S}\left(t\right))$$

The relevant region is {t:Pr⁡(D≠0|r(X)=t)≥Pr⁡(D≠0)} when the default, in the absence of risk prediction, is to take no action for any outcome.

Turning to the strong sense definitions, the key difference is that the predicted and actual outcomes must coincide for at least one outcome that did occur.

**Definition 15**

*Strong panel-wise sensitivity is the probability that at least one outcome is correctly predicted to occur in an individual for which at least one outcome did occur*.

$$sens_{P}\left(t\right)=\text{Pr}(D^{\text{′}}I(r(X)\geq t)\neq 0|D\neq 0)$$

Estimates of ${\text{sens}}_{P}\left(t\right)$ may be subject to ascertainment bias, which could be adjusted for by weighting each observation where D≠0 by the inverse of its sampling probability.

**Definition 16**

*Strong panel-wise specificity is the probability that all outcomes that did not occur are predicted not to occur in an individual for which at least one outcome did not occur*.

$$spec_{P}\left(t\right)=\text{Pr}({\left(1-D\right)}^{\text{′}}I\left(r\left(X\right)\geq t\right)=0|D\neq 1)$$

Definitions 15 and 16 complement each other in a different way to the weak sense definitions 15 and 16. The complement of strong panel-wise specificity is analogous to the strong sense of family-wise type-1 error in hypothesis testing. Note that an individual may count towards both sensitivity and specificity, a property shared with the outcome-wise measures.

**Definition 17**

*Strong panel-wise C-index is the probability that, given one individual in which at least one outcome did occur and one individual in which at least one outcome did not occur, the maximum risk prediction is greater among the outcomes that did occur in the former individual than among the outcomes that did not occur in the latter*.

$$C_{P}=\text{Pr}(\text{max}\left(D_{i_{1}}\circ r\left(X_{i_{1}}\right)\right)>\text{max}\left((1-D_{i_{0}})\circ r\left(X_{i_{0}}\right)\right)|D_{i_{1}}\neq 0,D_{i_{0}}\neq 1)$$

where ∘ denotes Hadamard product.

Note that under this definition an individual may appear on both sides of the inequality (i.e. $i_{1}=i_{0}$) and, unlike $C_{J}$ and $C_{S}$, $C_{P}$ does not have a natural interpretation as a measure of discrimination. Furthermore, it need not equal 0.5 under random predictions. Nevertheless it corresponds to definitions of sensitivity and specificity in the same way as those other measures of concordance, and could be used as a summary measure for comparing different predictors of a set of outcomes.

Relative utility cannot be developed in the same manner as $RU_{J}$ and $RU_{S}$, but the following working definition is analogous to that of the weak panel-wise sense.

**Definition 18**

*Strong panel-wise relative utility for threshold vector *t* is*

$$RU_{P}\left(t\right)=sens_{P}\left(t\right)-\frac{\;\mathrm{Pr}\left(D\neq 0\text{|}r\left(X\right)=t\right)}{\mathrm{Pr}\left(D\neq 1\text{|}r\left(X\right)=t\right)}\frac{\mathrm{Pr}\left(D\neq 1\right)}{\mathrm{Pr}\left(D\neq 0\right)}(1-spec_{P}\left(t\right))$$

with the relevant region

$$\left\{t:\frac{\mathrm{Pr}\left(D\neq 0\text{|}r\left(X\right)=t\right)}{\mathrm{Pr}\left(D\neq 1\text{|}r\left(X\right)=t\right)}\geq \frac{\mathrm{Pr}\left(D\neq 0\right)}{\mathrm{Pr}\left(D\neq 1\right)}\right\}$$

If risk predictions and outcomes both are jointly independent, and the risk predictor is weakly component-wise calibrated, then

$$RU_{P}\left(t\right)=sens_{P}\left(t\right)-\frac{1-\prod_{j}(1-t_{\left[j\right]})}{1-\prod_{j}t_{\left[j\right]}}\frac{\mathrm{Pr}\left(D\neq 1\right)}{\mathrm{Pr}\left(D\neq 0\right)}(1-spec_{P}\left(t\right))$$

Which of the weak or strong measures is more appropriate will depend on the application. For example, if the same action would be performed for all outcomes, it is less important to predict specific outcomes. That might be the case when screening for a range of conditions with a common intervention, as is done say when measuring blood pressure with a view to prescribing anti-hypertensives. For this reason I suggest *screening*, with subscript S, as a shorthand for weak panel-wise, and *panel-wise* itself, subscript P, as a shorthand for strong panel-wise, and will use those terms in the rest of the paper. (Strong) panel-wise measures may be appropriate in early detection settings where the action depends on the specific outcomes predicted.

Figure 1

**Figure 1.**
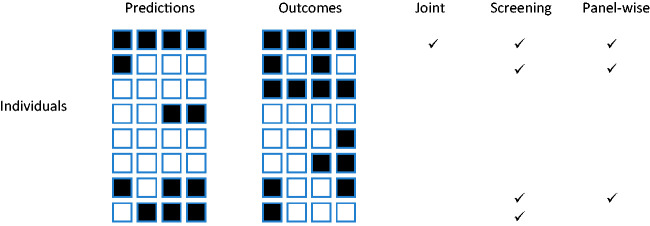
Example outcomes in eight individuals. Outcomes predicted to occur are shown in black on the left panel. Outcomes that did occur are shown in black on the right panel. Ticks show individuals counting in the numerator for each sense of sensitivity. Here the sample joint sensitivity is 1/2, the screening sensitivity is 4/7, and the panel-wise sensitivity is 3/7. The outcome-wise sensitivity is 7/16.

shows an example of four outcomes in eight individuals, showing which individuals count towards the different senses of sensitivity.

## 3 Multivariate probit model

For a single outcome, many measures of predictive accuracy can be expressed in terms of variance explained by the risk predictor, assuming a probit model for the outcome.^42^ This allows any of the measures to be derived from reported values of any others, and argues for the use of variance explained as a fundamental measure of prediction accuracy without the caveats associated with, for example, ROC curves. Here this framework is extended to the prediction of multiple traits using a multivariate probit model for outcomes.^43^

Assume that individual i has a latent *liability* vector $L_{i}$ distributed as multivariate normal with dimension m, mean vector 0 and variance–covariance matrix $\Sigma_{L}$ with diagonal entries 1. Define the threshold vector τ such that outcome j occurs whenever $L_{\left[j\right]}\geq \tau_{\left[j\right]}$, thus ${\text{τ}}_{\left[j\right]}={\text{Φ}}^{-1}(1-\mathrm{Pr}\left(D_{\left[j\right]}=1\right))$.

Assume that each outcome has a single normally distributed predictor, so that the predictor vector $X_{i}$ is multivariate normal with dimension m, mean vector 0 and variance–covariance matrix $\Sigma_{X}$. Let the joint distribution of liability and predictor be multivariate normal with mean vector 0 and variance–covariance matrix

$$\Sigma =\left[\begin{array}{cc} \Sigma_{L} & \Sigma_{LX}\\ \Sigma_{LX}^{\text{′}} & \Sigma_{X} \end{array}\right]$$

where component [jk] of $\Sigma_{LX}$ is the covariance between liability for outcome j and predictor of outcome k. A notable special case is $\Sigma_{LX}=\Sigma_{X}$. Then the diagonal elements of $\Sigma_{X}$ are the variances in each liability explained by the corresponding predictors, and for each outcome, conditional on its own predictor there is no additional information from any other predictors.

The following expressions will be useful. If each element of X estimates the corresponding element of L, the risk prediction for outcome j is given by

(1) $$r_{\left[j\right]}\left(X\right)=1-\text{Φ}\left(\frac{\tau_{\left[j\right]}-X_{\left[j\right]}}{\sqrt{1-{\text{Σ}}_{X\left[jj\right]}}}\right)$$

and the risk threshold $t_{\left[j\right]}$ is equivalent to the predictor threshold

$${\overset{˜t}{\;}}_{\left[j\right]}=\tau_{\left[j\right]}-{\left(1-{\text{Σ}}_{X\left[jj\right]}\right)}^{\frac{1}{2}}{\text{Φ}}^{-1}(1-t_{\left[j\right]})$$

Given outcomes D=d, the liability follows a multivariate truncated normal distribution, with truncation at τ from below for the outcomes that did occur and from above for those that did not. Denote the conditional mean vector and variance–covariance matrix of the truncated liability by $\mu_{L|D=d}$ and $\Sigma_{L|D=d}$; these quantities may be computed numerically by the method of Tallis.^44,45^ The Pearson-Aitken selection formulae^46^ give the mean predictor in individuals with outcomes d as

(2) $$E\left(X\text{|}D=d\right)=\Sigma_{LX}\Sigma_{L}^{-1}\mu_{L|D=d}$$

and the variance–covariance matrix

(3) $$var\left(X\text{|}D=d\right)=\Sigma_{X}-\Sigma_{LX}(\Sigma_{L}^{-1}-\Sigma_{L}^{-1}\Sigma_{L|D=d}\Sigma_{L}^{-1})\Sigma_{LX}^{\text{′}}$$

Assume that conditional on d the predictor follows the m-variate normal distribution with the above mean and variance–covariance. Furthermore since L has mean 0

$$\mu_{L|D\neq d}=-\frac{\mathrm{Pr}\left(D=d\right)}{\mathrm{Pr}\left(D\neq d\right)}\mu_{L|D=d}$$

and

$$\Sigma_{L|D\neq d}=\frac{\Sigma_{L}-\text{Pr}(D=d)\Sigma_{L|D=d}}{1-\text{Pr}(D=d)}-{\left[\frac{\text{Pr}(D=d)}{1-\text{Pr}(D=d)}\right]}^{2}\mu_{L|D=d}^{\text{′}}\mu_{L|D=d}$$

from which E(X|D≠d) and var(X|D≠d) follow analogously to equations (2) and (3).

Finally assume that conditional on a prediction X=x the liability follows the m-variate normal distribution with the mean and variance–covariance given by the Pearson-Aitken selection formulae as

(4) $$E\left(L\text{|}X=x\right)=\Sigma_{LX}\Sigma_{X}^{-1}x$$

(5) $$var\left(L\text{|}X=x\right)=\Sigma_{L}-\Sigma_{LX}\Sigma_{X}^{-1}\Sigma_{LX}^{\text{′}}$$

The outcome-wise criteria can be expressed in terms of single outcome criteria, which are special cases of the joint criteria below and are therefore omitted for brevity.

### 3.1 Joint criteria

From Definition 7

sensJ(t)=Pr(r(X)≥t|D=1)=Pr(r(X)≥t,D=1)Pr(D=1)=Φ((−τ,− ˜t)′;0,Σ)Φ(−τ;0,ΣL)

where Φ(·,μ,Σ) denotes the multivariate normal cumulative distribution function with mean vector μ and variance–covariance matrix Σ.

From Definition 8

specJ(t)=Pr(I(r(X)≥t)≠1|D≠1)=1−Pr(I(r(X)≥t)=1,D≠1)Pr(D≠1)=1−Pr(I(r(X)≥t)=1)−Pr(I(r(X)≥t)=1,D=1)1−Pr(D=1)=1−Φ(−˜t;0,ΣX)−Φ((−τ,− ˜t)′;0,Σ)1−Φ(−τ;0,ΣL)

Calculating joint concordance requires the distribution of the maximum element of the multivariate risk predictor. This has recently been derived analytically^47^ but can be approximated by simulation. First simulate a predictor from the multivariate normal distribution conditional on D=1, given by equations (2) and (3), and convert each component to a risk using equation (1). Simulate a second predictor in the same way but conditional on D≠1. Over a large number of simulations, the joint concordance is estimated as the proportion in which the minimum risk of the first predictor exceeds the minimum in the second.

From Definition 10, the joint relative utility is

$$RU_{J}\left(t\right)=sens_{J}\left(t\right)-\frac{\;\mathrm{Pr}\left(D=1\text{|}r\left(X\right)=t\right)}{\mathrm{Pr}\left(D\neq 1\text{|}r\left(X\right)=t\right)}\frac{\mathrm{Pr}\left(D\neq 1\right)}{\mathrm{Pr}\left(D=1\right)}(1-spec_{J}\left(t\right))$$

with $\mathrm{Pr}\left(D=1\right)=\Phi (-\tau ;0,\Sigma_{L})$, and $\mathrm{Pr}\left(D=1\text{|}r\left(X\right)=t\right)=\Phi (-\tau ;-\mu_{L|X=t},\Sigma_{L|X=t})$ where $\mu_{L|X=t}$ and $\Sigma_{L|X=t}$ are given by equations (4) and (5), respectively.

### 3.2 Screening criteria

Following analogous steps to the joint measures, from Definition 11

$$sens_{S}\left(t\right)=1-\frac{\Phi \left(\overset{˜t}{\;};0,\Sigma_{X}\right)-\Phi ({\left(\tau ,\overset{˜t}{\;}\right)}^{\text{′}};0,\Sigma )}{1-\Phi \left(\tau ;0,\Sigma_{L}\right)}$$

From Definition 12

$$spec_{S}\left(t\right)=\frac{\Phi ({\left(\tau ,\overset{˜t}{\;}\right)}^{\text{′}};0,\Sigma )}{\Phi \left(\tau ;0,\Sigma_{L}\right)}$$

To estimate screening concordance, first simulate a predictor from the multivariate normal distribution conditional on D=0, given by equations (2) and (3), and convert each component to a risk using equation (1). Simulate a second predictor in the same way but conditional on D≠0. Over a large number of simulations, the screening concordance is estimated as the proportion in which the maximum risk of the second predictor exceeds the maximum in the first.

From definition 14, the screening relative utility is

$$RU_{S}\left(t\right)=sens_{S}\left(t\right)-\frac{\;\mathrm{Pr}\left(D\neq 0\text{|}r\left(X\right)=t\right)}{\mathrm{Pr}\left(D=0\text{|}r\left(X\right)=t\right)}\frac{\mathrm{Pr}\left(D=0\right)}{\mathrm{Pr}\left(D\neq 0\right)}(1-spec_{S}\left(t\right))$$

with $\mathrm{Pr}\left(D=0\right)=\Phi (\tau ;0,\Sigma_{L})$, and $\mathrm{Pr}\left(D=0\text{|}r\left(X\right)=t\right)=\Phi (\tau ;\mu_{L|X=t},\Sigma_{L|X=t})$.

### 3.3 Panel-wise criteria

Panel-wise measures can be evaluated by summing over outcome vectors d. From Definition 15 the panel-wise sensitivity is

sensP(t)=Pr(D′I(r(X)≥t)≠0|D≠0)=1−Pr(D′I(r(X)≥t)=0|D≠0)=1−∑d:d≠0Pr(D′I(r(X)≥t)=0|D=d)Pr(D=d|D≠0)=1−11−Φ(τ;0,ΣL)∑d:d≠0Pr(D′I(r(X)≥t)=0,D=d)

The probability in the summand is an integral of the multivariate normal density with mean vector **0** and variance–covariance matrix Σ. For components j where $d_{\left[j\right]}=1$, the limits of integration are $\left[{\text{τ}}_{\left[j\right]},\infty \right]$ for the liability components and $\left[-\infty ,{\overset{˜t}{\;}}_{\left[j\right]}\right)$ for the predictor components. For components j where $d_{\left[j\right]}=0$, the limits are $\left[-\infty ,\tau_{\left[j\right]}\right)$ and [−∞,∞], respectively.

From definition 16 the panel-wise specificity is

specP(t)=Pr((1−D)′I(r(X)≥t)=0|D≠1)=11−Φ(−τ;0,ΣL)∑d:d≠1Pr((1−D)′I(r(X)≥t)=0,D=d)

For components j where $d_{\left[j\right]}=1$, the limits of integration are $[\tau_{\left[j\right]},\infty$] for the liability components and [−∞,∞] for the predictor components. For components j where $d_{\left[j\right]}=0$, the limits are $\left[-\infty ,\tau_{\left[j\right]}\right)$ and $\left[-\infty ,{\overset{˜t}{\;}}_{\left[j\right]}\right),$ respectively.

To estimate panel-wise concordance, simulate liabilities L and predictors X from their joint multivariate normal distribution with mean vector 0 and variance–covariance matrix Σ. Concordance is estimated according to Definition 17 using pairs of simulated L and X in which one has D≠1 and the other has D≠0.

The panel-wise relative utility can be calculated from Definition 18 using expressions given above.

All the criteria are now expressed in terms of the marginal outcome probabilities $\mathrm{Pr}\left(D_{\left[j\right]}=1\right)$ and the joint variance–covariance matrix Σ of liability and predictor. A summary measure of the prediction accuracy is suggested by the multivariate analysis of variance, via Wilks’ Λ

$$1-\text{Λ}=1-\frac{\text{det}(\Sigma_{L}+\Sigma_{X}-\Sigma_{LX}-\Sigma_{LX}^{\text{′}})}{\text{det}(\Sigma_{L})}$$

This is the proportion of variance of L explained by the predictor X. For a single outcome, 1−Λ equals the coefficient of determination from the regression of L on X.^42^

## 4 Examples

### 4.1 CancerSEEK

CancerSEEK is a blood-based test of circulating proteins and tumour DNA mutations that are associated with the presence of cancer.^9^ It has been proposed for early detection of cancers of the ovary, liver, stomach, pancreas, esophagus, colorectum, lung, or breast. A single test is applied, from which a positive result suggests the presence of one of these cancers. Given a positive test, a secondary algorithm identifies the likely site of the cancer.

CancerSEEK tests a composite outcome, and as such the standard univariate criteria correspond to screening criteria. However, the authors reported sensitivities for each cancer individually, at a risk threshold of 0.893, and reported their incidence-weighted average as 55%. This average corresponds to outcome-wise sensitivity (Definition 3), but it is also a screening sensitivity if at most one cancer is present in each subject. The screening specificity was reported as over 99%.

The in-sample screening sensitivity at this risk threshold was 62.2% and the area under the ROC curve (AUC) was 91% (Figure 2a in Cohen et al.^9^). However, as noted in Definition 11 these estimates are subject to ascertainment bias, in particular the under-sampling of breast cancers relative to other cancer cases, explaining the discrepancy between the in-sample and incidence-weighted sensitivities. I randomly resampled cases from each cancer (their Table S4) in proportion to their incidence rates (L. Danilova, personal communication). The in-sample screening sensitivity was now 55%, equal to the outcome-wise sensitivity, and the screening concordance reduced to 89%. This is the concordance that would be expected in a population screening context.

### 4.2 Polygenic risk scores

A polygenic risk score (PRS) is an aggregation of genetic risk, ${\hat{\beta }}^{\text{′}}G$ where $\hat{\beta }$ is a vector of estimated effects (e.g. log odds ratios) and G is a vector of coded genotypes (e.g. numbers of risk alleles) across many DNA sites, typically single nucleotide polymorphisms (SNPs).^48^ A PRS can be computed for many diseases at once in the same individual, by forming products of different effect vectors with the fixed genotype vector.

PRS have been constructed for a number of diseases and have shown promise for risk prediction.^10^ Table 1

**Table 1.** Properties of fitted PRS for six common diseases.

Disease	AUC	Prevalence	Liability $R^{2}$	SNP $h^{2}$	Sensitivity	Specificity
Type-2 Diabetes	0.66	0.102	0.0856	0.196	0.630	0.599
Coronary Artery Disease	0.623	0.0461	0.0398	0.22	0.600	0.575
Crohn’s Disease	0.75	0.005	0.103	0.26	0.701	0.666
Ulcerative Colitis	0.7	0.0025	0.0553	0.19	0.657	0.632
Schizophrenia	0.62	0.01	0.0254	0.235	0.595	0.576
Rheumatoid Arthritis	0.7	0.01	0.0732	0.18	0.661	0.629

Note: AUC and Prevalence, the reported values in the literature.^54–60^ Liability ${\text{R}}^{2}$, the diagonal elements of ${\text{Σ}}_{\text{LX}}$ derived from AUC and Prevalence ^49^. SNP $h^{2}$, the liability variance explained by all genotyped SNPs, which is the maximum possible value of Liability $R^{2}$.^24,60–63^ Sensitivity and sensitivity, their values when risk threshold equals the prevalence.

shows six diseases for which PRS have been fitted using variants across the whole genome, as opposed to a limited number of associated SNPs. The reported AUCs were converted to liability variances explained using published formulae,^49^ giving the diagonal elements of $\Sigma_{LX}$. Assume that the correlation between pairs of estimated PRS equals the total genetic correlation of the diseases, which was obtained from the LD-Hub database^50^ (Table 2

**Table 2.** Variance–covariance matrix ${\text{Σ}}_{X}$ between PRS for the six diseases of Table 1.

	T2D	CAD	CD	UC	SCZ	RA
T2D	0.0856					
CAD	0.0225	0.0398				
CD	−0.0111	0.0347	0.102			
UC	−0.0086	0.0191	0.0409	0.0553		
SCZ	−0.00131	0	0.00679	0.00480	0.0254	
RA	−0.038	−0.034	−0.00251	0.00566	−0.00185	0.0732

Note: Assumed to equal the liability-PRS covariance matrix ${\text{Σ}}_{\text{LX}}$.

) to give the off-diagonal elements of $\Sigma_{X}$. This assumption is more tenable for these PRS, which include variants across the whole genome, than for PRS constructed from a limited number of associated SNPs. Assume further that the correlation between disease liabilities also equals the genetic correlation, giving $\Sigma_{L}$ (Table 3

**Table 3.** Genetic correlations between the six diseases of Table 1.

	T2D	CAD	CD	UC	SCZ	RA
T2D	1					
CAD	0.384	1				
CD	−0.119	0.057	1			
UC	−0.125	0.038	0.543	1		
SCZ	−0.028	0	0.113	0.128	1	
RA	−0.048	−0.063	−0.029	0.089	−0.043	1

Note: Assumed to equal the correlations between their overall liabilities ${\text{Σ}}_{L}$.

). Finally assume that the PRS for disease j has no covariance with disease liability k conditional on the PRS for disease k, where j≠k. Under this assumption $\Sigma_{X}=\Sigma_{LX}$ (Table 2).

Under the model developed in section 3, the event-wise concordance is 0.653, the screening concordance is 0.607, which is lower than all individual AUCs, and the joint concordance is 0.749. The panel-wise concordance is 0.49, compared to a value of 0.37 obtained when the correlation matrices are the same but all individual AUCs are set to 0.5.

For illustration, consider a screening application to identify, early in life, those at elevated risk of at least one of these diseases. Suppose the risk threshold vector is set equal to the prevalence, so that the predictor identifies individuals with above-average predicted risk for at least one disease. The screening sensitivity is 0.955, which is considerably higher that the individual sensitivities (Table 1). However, the screening specificity is much lower at 0.074. Similarly to multiple hypothesis testing, the prediction of multiple outcomes increases both the true-positive and false-positive rate at a given threshold vector, but the thresholds that reflect the cost–benefit ratio are different in the multiple prediction context than for the single predictions. The screening concordance of 0.607 suggests that, across all thresholds regarded equally, the sensitivity-specificity trade-off is not as good as for any disease individually. The screening relative utility is −0.004, suggesting that these PRS provide no benefit in a multiple screening application. The liability variance explained is 1−Λ = 0.332, which of itself is higher than the individual $R^{2}$ (Table 1) but, as just seen, leads to lower values of several criteria of accuracy.

In principle, PRS could be developed that explain greater proportions of liability^48^ up to the so-called SNP heritability (Table 1). Under this scenario the liability variance explained increases to 1−Λ = 0.765, giving a screening concordance of 0.664 and relative utility of 0.275. This suggests that further progress in genetic prediction may lead to more useful applications in multiple screening contexts, especially if further combined with non-genetic risk factors.

## 5 Discussion

Standard concepts of sensitivity and specificity generalise naturally to the multivariate setting. Positive and negative predictive values generalise similarly, and for completeness their definitions are provided in the supplementary text. Although the ROC curve does not extend so easily, the related concept of concordance does so. However, in contrast to the single outcome setting, concordance is sensitive to the outcome probabilities, negating one perceived advantage of that criterion. In the strong panel-wise sense the concordance is unsatisfying because an individual can be regarded as being discordant with itself, and there is no natural interpretation in terms of discrimination. The range of panel-wise concordance depends upon the number of outcomes and the covariance of predictors and outcomes, and may fall below 0.5. In practice its minimum value can be estimated by simulation or theory, as in section 4.2, by setting the predictors to be independent of the outcomes while maintaining the correlation among predictors and among outcomes. Strong panel-wise measures have an intermediate position between outcome-wise and screening measures, in that prediction is evaluated at the individual level but the predictions of specific outcomes are taken into account. The proposed definitions are motivated by possible applications in early detection of disease, and have convenient analogies with family-wise error in hypothesis testing, but other approaches may be possible.

Relative utility, which is a useful summary of sensitivity and specificity when predicting a single outcome, presents some difficulties when predicting multiple outcomes. I propose definitions assuming common benefits and costs for all outcomes, which allow analogous development to that for a single outcome, but may lead to sub-optimal assessment of utility when the benefits and costs vary across outcomes. When outcomes are correlated, accurate calculation of relative utility may be difficult, so approximations are provided assuming independent predictors and outcomes. It remains to be seen how useful these definitions prove in practice, given their assumptions of common additive benefits and costs, and independent predictors and outcomes.

Some examples of screening have been discussed, but examples of outcome-wise or joint accuracy can also be envisaged. CancerSEEK is a recent example of molecular technology applied to early detection of multiple cancers. Its performance was reported in the screening sense, but the proposed definitions clarify that all quantities can be affected by ascertainment bias. The present criteria are more sensitive to incidence and sampling rates than the corresponding univariate measures.

I have only considered the accuracy of a given predictor, and have not considered how such predictors are constructed. Multivariate predictors could be constructed simply by concatenating univariate predictors. The example of PRS shows that this is feasible and pragmatic given that such scores are currently constructed from case/control studies of individual diseases. In future, given the increasing availability of extensive phenotyping in large cohorts, it will be possible to build prediction models with the optimisation of multiple outcome prediction as the direct objective. Methodology for such model building is a fertile area for future work.

Prediction models are often evaluated for their improvement over existing models. Evaluation of incremental performance remains a controversial subject when predicting a single trait. Among several proposed measures the net reclassification index has attained a default status among practitioners yet has received strong criticism.^51,52^ Such issues are likely to be magnified when predicting multiple traits.

Given predictors for a set of outcomes, a natural question is whether there is some subset of outcomes for which risk prediction is most effective. Naïve comparison of, say, relative utilities for different groups of outcomes would be inappropriate without consideration of the relative benefits of predicting each group. Thus, the finding that the screening concordance of PRS is lower over six diseases than for each disease individually should not in itself argue against a screening application, because the benefits and costs of screening six diseases are different from those of screening one disease. Many authors have argued for decision-theoretic treatments of risk prediction.^28,53^ Such approaches can also be developed for the multiple outcome setting and would put the comparison of predictors for different groups of outcomes on a more coherent footing.

Competing risks present a problem for mutually exclusive outcomes, such as diseases of later life. There is a distinction between accounting for competing risks in model building, and in model evaluation. The emphasis here is on evaluation, for which the proposed criteria could be adapted to account for competing risks. However, the explicit consideration of multiple outcomes may encourage more careful consideration of competing risks at the model building stage and lead to improved prediction in general.

An R library to calculate these criteria from empirical data, and to evaluate the multivariate probit formulae of section 3, is available from https://github.com/DudbridgeLab/multipred.

## Supplemental Material

sj-pdf-1-smm-10.1177_0962280220929039 - Supplemental material for Criteria for evaluating risk prediction of multiple outcomes Click here for additional data file. Supplemental material, sj-pdf-1-smm-10.1177_0962280220929039 for Criteria for evaluating risk prediction of multiple outcomes by Frank Dudbridge in Statistical Methods in Medical Research
