# Supplementary material for: Criteria for evaluating risk prediction of multiple outcomes
Source: Stat Methods Med Res. 2020 Jun 29;29(12):3492–510. doi: 10.1177/0962280220929039 (PMC7682512; doi:10.1177/0962280220929039)
Supplement: sj-pdf-1-smm-10.1177_0962280220929039 - Supplemental material for Criteria for evaluating risk prediction of multiple outcomes [file sj-pdf-1-smm-10.1177_0962280220929039.pdf]

Criteria for evaluating risk prediction of multiple outcomes

Supplementary text

Positive and negative predictive value are defined similarly to sensitivity and specificity, with a change in the denominator.

Definition S1. Outcome-wise positive predictive value is the probability of an outcome occurring given that it was predicted,

$$PPV_O(\boldsymbol{t}) = \frac{E_{\boldsymbol{D},\boldsymbol{X}}[\boldsymbol{D}'\boldsymbol{I}(r(\boldsymbol{X}) \geq \boldsymbol{t}])}{E_{\boldsymbol{X}}[\mathbf{1}'\boldsymbol{I}(r(\boldsymbol{X}) \geq \boldsymbol{t}))]}$$

It is the weighted sum of the individual outcome positive predictive values, with the weights as the relative probabilities of positive predictions. General weights may be introduced.

Definition S2. Outcome-wise negative predictive value is the probability of an outcome not occurring given that it was not predicted,

$$NPV_O(\boldsymbol{t}) = \frac{E_{\boldsymbol{D},\boldsymbol{X}}[(\mathbf{1} - \boldsymbol{D})'\boldsymbol{I}(r(\boldsymbol{X}) < \boldsymbol{t}])}{E_{\boldsymbol{X}}[\mathbf{1}'\boldsymbol{I}(r(\boldsymbol{X}) < \boldsymbol{t}))]}$$

It is the weighted sum of the individual outcome negative predictive values, with the weights as the relative probabilities of negative predictions. General weights may be introduced.

Definition S3. Joint positive predictive value is the probability of all outcomes occurring, in an individual for which all outcomes are predicted to occur,

$$PPV_J(\boldsymbol{t}) = \Pr\left(\boldsymbol{D} = \mathbf{1} | r(\boldsymbol{X}) \geq \boldsymbol{t}\right)$$

In the case that the risk predictions and outcomes both are jointly independent, the joint positive predictive value is the product of the individual outcome positive predictive values. Under the multivariate probit model of section 3,

$$PPV_J(\boldsymbol{t}) = \frac{\boldsymbol{\Phi}((-\boldsymbol{\tau}, -\tilde{\boldsymbol{t}})'; \mathbf{0}, \boldsymbol{\Sigma})}{\boldsymbol{\Phi}(-\tilde{\boldsymbol{t}}; \mathbf{0}, \boldsymbol{\Sigma}_X)}$$

Definition S4. Joint negative predictive value is the probability of at least one outcome not occurring, in an individual for which at least one outcome is predicted not to occur,

$$NPV_J(\boldsymbol{t}) = \Pr\left(\boldsymbol{D} \neq \boldsymbol{1} | \boldsymbol{I}(r(\boldsymbol{X}) \geq \boldsymbol{t}) \neq \boldsymbol{1}\right)$$

Under the multivariate probit model,

$$NPV_J(\boldsymbol{t}) = 1 - \frac{\boldsymbol{\Phi}(-\boldsymbol{\tau}; \boldsymbol{0}, \boldsymbol{\Sigma}_L) - \boldsymbol{\Phi}((-\boldsymbol{\tau}, -\tilde{\boldsymbol{t}})'; \boldsymbol{0}, \boldsymbol{\Sigma})}{1 - \boldsymbol{\Phi}(-\tilde{\boldsymbol{t}}; \boldsymbol{0}, \boldsymbol{\Sigma}_X)}$$

Definition S5. Weak panel-wise positive predictive value is the probability of at least one outcome occurring, in an individual for which at least one outcome is predicted to occur,

$$PPV_S(\boldsymbol{t}) = \Pr\left(\boldsymbol{D} \neq \boldsymbol{0} | \boldsymbol{I}(r(\boldsymbol{X}) \geq \boldsymbol{t}) \neq \boldsymbol{0}\right)$$

Under the multivariate probit model,

$$PPV_S(\boldsymbol{t}) = 1 - \frac{\boldsymbol{\Phi}(\boldsymbol{\tau}; \boldsymbol{0}, \boldsymbol{\Sigma}_L) - \boldsymbol{\Phi}((\boldsymbol{\tau}, \tilde{\boldsymbol{t}})'; \boldsymbol{0}, \boldsymbol{\Sigma})}{1 - \boldsymbol{\Phi}(\tilde{\boldsymbol{t}}; \boldsymbol{0}, \boldsymbol{\Sigma}_X)}$$

Definition S6. Weak panel-wise negative predictive value is the probability of no outcomes occurring, in an individual for which no outcomes are predicted to occur,

$$NPV_S(\boldsymbol{t}) = \Pr\left(\boldsymbol{D} = \boldsymbol{0} | r(\boldsymbol{X}) < \boldsymbol{t}\right)$$

In the case that the risk predictions and outcomes both are jointly independent, the weak panel-wise negative predictive value is the product of the individual outcome negative predictive values. Under the multivariate probit model,

$$NPV_S(\boldsymbol{t}) = \frac{\boldsymbol{\Phi}((\boldsymbol{\tau}, \tilde{\boldsymbol{t}})'; \boldsymbol{0}, \boldsymbol{\Sigma})}{\boldsymbol{\Phi}(\tilde{\boldsymbol{t}}; \boldsymbol{0}, \boldsymbol{\Sigma}_X)}$$

Definition S7. Strong panel-wise positive predictive value is the probability of at least one outcome occurring that is predicted to occur, in an individual for which at least one outcome is predicted to occur,

$$PPV_F(\boldsymbol{t}) = \Pr\left(\boldsymbol{D}'\boldsymbol{I}(r(\boldsymbol{X}) \geq \boldsymbol{t}) \neq \boldsymbol{0} \middle| \boldsymbol{I}(r(\boldsymbol{X}) \geq \boldsymbol{t}) \neq \boldsymbol{0}\right)$$

Under the multivariate probit model,

$$PPV_F(\boldsymbol{t}) = \Pr\left(\boldsymbol{D}'\boldsymbol{I}(r(\boldsymbol{X}) \geq \boldsymbol{t}) \neq \boldsymbol{0} \middle| \boldsymbol{I}(r(\boldsymbol{X}) \geq \boldsymbol{t}) \neq \boldsymbol{0}\right)$$

$$= 1 - \frac{1}{1 - \boldsymbol{\Phi}(\tilde{\boldsymbol{t}}; \boldsymbol{0}, \boldsymbol{\Sigma}_X)} \sum_{\boldsymbol{x}:\boldsymbol{I}(r(\boldsymbol{x}) \geq \boldsymbol{t}) \neq \boldsymbol{0}} \Pr\left(\boldsymbol{D}'\boldsymbol{I}(r(\boldsymbol{X}) \geq \boldsymbol{t}) = \boldsymbol{0}, \boldsymbol{I}(r(\boldsymbol{X}) \geq \boldsymbol{t}) = \boldsymbol{x}\right)$$

The probability in the summand is an integral of the multivariate normal density with mean vector $\boldsymbol{0}$ and variance-covariance matrix $\boldsymbol{\Sigma}$. For components j where $r_{[j]}(\boldsymbol{x}) \geq t_{[j]}$, the limits of integration are $[-\infty, \tau_{[j]})$ for the liability components and $[\tilde{t}_{[j]}, \infty]$ for the predictor components. For components j where $r_{[j]}(\boldsymbol{x}) < t_{[j]}$, the limits are $[-\infty, \infty]$ and $[-\infty, \tilde{t}_{[j]})$ respectively.

Definition S8. Strong panel-wise negative predictive value is the probability of all outcomes not occurring that are predicted not to occur, in an individual for which at least one outcome is predicted not to occur,

$$NPV_F(\boldsymbol{t}) = \Pr\left(\boldsymbol{D}'\boldsymbol{I}(r(\boldsymbol{X}) < \boldsymbol{t}) = \boldsymbol{0} \middle| \boldsymbol{I}(r(\boldsymbol{X}) \geq \boldsymbol{t}) \neq \boldsymbol{1}\right)$$

Under the multivariate probit model,

$$NPV_F(\boldsymbol{t}) = \Pr\left(\boldsymbol{D}'\boldsymbol{I}(r(\boldsymbol{X}) < \boldsymbol{t}) = \boldsymbol{0} \middle| \boldsymbol{I}(r(\boldsymbol{X}) \geq \boldsymbol{t}) \neq \boldsymbol{1}\right)$$

$$= \frac{1}{1 - \boldsymbol{\Phi}(-\tilde{\boldsymbol{t}}; \boldsymbol{0}, \boldsymbol{\Sigma}_X)} \sum_{\boldsymbol{x}:\boldsymbol{I}(r(\boldsymbol{x}) \geq \boldsymbol{t}) \neq \boldsymbol{1}} \Pr\left(\boldsymbol{D}'\boldsymbol{I}(r(\boldsymbol{X}) < \boldsymbol{t}) = \boldsymbol{0}, \boldsymbol{I}(r(\boldsymbol{X}) \geq \boldsymbol{t}) = \boldsymbol{x}\right)$$

For components j where $r_{[j]}(\boldsymbol{x}) \geq t_{[j]}$, the limits of integration are $[-\infty, \infty]$ for the liability components and $[\tilde{t}_{[j]}, \infty]$ for the predictor components. For components j where $r_{[j]}(\boldsymbol{x}) < t_{[j]}$, the limits are $[-\infty, \tau_{[j]})$ and $[-\infty, \tilde{t}_{[j]})$ respectively.
